# Anti-Inflammatory properties of Salograviolide A purified from Lebanese plant *Centaurea ainetensis*

**DOI:** 10.1186/1472-6882-9-36

**Published:** 2009-09-23

**Authors:** Jamal Al-Saghir, Randa Al-Ashi, Ralph Salloum, Najat A Saliba, Rabih S Talhouk, Fadia R Homaidan

**Affiliations:** 1 Center for Biodiversity Studies, Interdisciplinary Biodiversity Studies in Arid Regions (IBSAR); American University of Beirut, Beirut, Lebanon; 2 Department of Physiology, Faculty of Medicine, American University of Beirut, Beirut, Lebanon; 3 Department of Chemistry, Faculty of Arts and Sciences, American University of Beirut, Beirut, Lebanon; 4 Department of Biology, Faculty of Arts and Sciences, American University of Beirut, Beirut, Lebanon; 5 Department of Internal Medicine/Pediatrics, School of Medicine, Wayne State University, Detroit, Michigan 48201, USA

## Abstract

**Background:**

Anti-inflammatory activities of medicinal plants have largely been attributed to their content of sesquiterpene lactones (SLs). SLs are predominantly found in the sunflower family *Asteraceae *and have been isolated from many plants of this family, particularly *Centaurea*. The anti-inflammatory activities of extract of *Centaurea ainetensis*, a Lebanese endemic plant, and the isolated active molecule were assessed for their potential ant-inflammatory activities.

**Methods:**

Plant extract from *Centaurea ainetensis*, and the isolated active ingredient Salograviolide A (SA), a sesquiterpene lactones guaianolide, were used for the study. Western blotting and electrophoretic mobility shift assays were used to test the effects of the plant extract and SA on interleukin-1 (IL-1) induced increase in cyclooxygenase-2 (COX-2) levels and in nuclear factor-κB (NF-κB) translocation in an intestinal epithelial cell (IEC) of inflammation. Their effects on inflammation score and cytokine levels were also studied in an iodoacetoamide-induced rat model of inflammation.

**Results:**

Plant extract and SA were shown to reverse the effects observed by IL-1 on COX-2 levels and NF-κB translocation in IEC. SA decreased the level of inflammatory cytokines and the level of inflammation in the animal model.

**Conclusion:**

These findings suggest that SA may be useful in the development of natural therapies for inflammatory diseases.

## 1. Background

Inflammatory bowel disease (IBD) is represented by a group of inflammatory conditions affecting the mucosa of the small intestine or colon. Immune activation and the inflammatory response in the intestine, as in other organs, are regulated by cytokines and other mediators of inflammation. These mediators include cytokines such as Interleukin-1 (IL-1),-6, and TNF-α, and others substances such as prostaglandins and leukotrienes [1].

IL-1, a pro-inflammatory cytokine, is produced by many inflammatory cell types in response to a variety of stimuli [2]. It has been shown to be increased in the intestinal mucosa of IBD patients and in animal models of intestinal inflammation [3]. We have shown that, in intestinal epithelial cells (IECs), IL-1 induced the synthesis of the enzyme cyclooxygenase-2 (COX-2) through the activation and translocation of the transcription factor, nuclear factor kappa B (NF-κB) [4]. NF-κB is most frequently composed of a p50 and a p65 subunit and under basal conditions it is retained in the cytoplasm bound to an inhibitory subunit IκB. In response to inflammatory stimulators, p65 subunit dissociates from IκB subunit and translocates from the cytoplasm to the nucleus, where it dimerizes with the p50 subunit and interacts with specific target genes, such as COX-2 leading to increased inflammatory processes [5,6]. Because of its central role in regulating inflammatory responses, a pharmacological inhibition of NF-κB activation could be beneficial in the treatment of inflammation [7].

Interest in alternatives to modern medicine has never been higher than it is now, and a large part of this interest revolves around the use of medicinal plants. Many of the anti-inflammatory activities of some medicinal plants were attributed to their contents of sesquiterpene lactones (SLs) [8-13]. In folk medicine, a diversity of plants, containing SLs, were used orally for the treatment of fever, hepatitis, bronchitis, malaria, viral infections, and topically for wounds, hematomas, sprains and rheumatic diseases [8-13]. Several studies investigated how these natural compounds exert their anti-inflammatory effects. SLs was shown to decrease inflammatory mediators such as IL-1β and TNF-α [14], prostaglandin E_2 _(PGE_2_) [15], nitric oxide (NO) [16,17], histamine and serotonin [18,19]; down-regulate the expression of major inflammatory enzymes such as cyclooxygenase-2 (COX-2) [15,20], 5-lipoxygenase (LOX) [21], and inducible nitric oxide synthase (iNOS) [17]; and decrease the DNA binding activity of the transcription factor NF-κB [20,22]. The anti-inflammatory action of SLs was also confirmed *in vivo *in acute murine ear [23] and paw edema [24] assays as well as chronic mouse ear edema models [25]. These activities were suggested to be mediated chemically through the action of α,β-unsaturated carbonyl structures, such as an α-methylene-γ-lactone or an α,β-unsubstituted cyclopentenone. These structure elements can react with nucleophiles, especially cysteine sulfhydryl groups, *via *a Michael-type addition [26,27]. Exposed thiol groups, such as cysteine residues in proteins, thus appear to be the primary targets of sesquiterpene lactones. SLs can be traced to a common biosynthetic pathway that starts with the cyclization of farnesyl or nerolidyl pyrophosphates. This is followed by oxidation and formation of the lactone leading to the synthesis of germacranolides SL. Following further ring closure, germacranolides can give rise to santanolides, eudesmanolides or guaianolides which are consequently considered to be the precursors of other classes of SLs [28].

SLs are found predominantly in the sunflower family Asteraceae (Compositae) and have been isolated in many plants of this family and particularly *Centaurea*, one of the largest genera [29-32]. *Centaurea ainetensis*, a Lebanese endemic plant that grows in stony usually sterile places, was reported previously by us to possess anti-fungal activities [33]. The plants was identified to the genus and specie level by Dr Stephen Jury "Royal Botanic Garden, Kew, West Sussex, London UK.

A crude decoction plant extract from *Centaurea ainetensis *was prepared and was used in studying potential anti-inflammatory activities. Further bioguided fractionation procedure [34] allowed the isolation and identification of the guaianolide, Salograviolide A (SA). SA has been also isolated from the aerial parts of another *Centaurea *species, *Centaurea Nicolai*, and has been shown to possess anti-fungal activity confirmed by *in-vitro *susceptibility assays [35]. In the present study the anti-inflammatory activities of the extract of *Centaurea ainetensis *and the isolated molecule Salograviolide A were investigated. The effects of the extract and SA on COX-2 expression and NF-κB translocation in an intestinal epithelial cell model of inflammation were studied. Furthermore, SA ability to reverse and/or prevent inflammation in a rat model of IBD was also evaluated.

## 2. Methods

### 2.1 Materials

All deuterated and non deuterated solvents were purchased from ACROS ORGANICS, Belgium and the preparative TLC plates, silica cartridges and silica powder were obtained from Alltech Associates, PA, USA. Fetal Bovine Serum (FBS), bovine serum albumin, Dulbecco's Modified Eagle's Medium (DMEM), Non-essential amino acids, penicillin and streptomycin, trypsin-EDTA were purchased from Invitrogen (Carlsbad, CA, USA). Human recombinant Interleukin-1α was from U.S. Biological (Cleveland, OH, USA). Rabbit polyclonal COX-2 antibody was from Cayman Chemicals (Michigan, USA). Polyvinylidene difluoride (PVDF) Hi-bond membranes, poly-dIdC, poly-dN6, Sodium Dodecyl Sulfate (SDS), glycine, Tris, glycerol, 2β-mercaptoethanol and γ-^32^P ATP were from Amersham Biosciences (San Diego, CA, USA). Protein Determination kit, Acrylamide and N', N'-bis-methylene acrylamide were from BioRad (Hercules, CA, USA). N,N,N',N',tetramethylethylenediamine (TEMED), ammonium persulfate (APS), ethylenediamine tetraacetic acid (EDTA), methanol, acetic acid, isopropanol, and dithiothreitol were from Sigma (St. Louis, MO, USA). Protease Inhibitor cocktail was from Biomol (Plymouth Meeting, PA, USA). Rabbit polyclonal antisera to IκB-α, NF-κB consensus oligonucleotide, Western Blotting Luminol reagents, ECL marker and horse radish peroxidase (HRP) conjugated secondary antibodies were purchased from Santa Cruz Biotechnology (Santa Cruz, CA, USA).

### 2.2 Methods

For testing the biological activity of *Centaurea ainetensis*, a crude decoction plant extract was prepared and was used in this study.

#### 2.2.1 Isolation of Salograviolide A from Centaurea ainetensis

**Figure 1:**
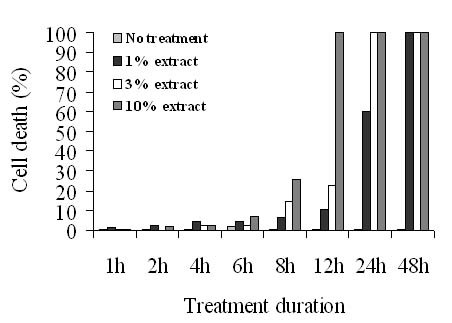
**Trypan blue exclusion assay on Mode-K cells treated with different concentrations of plant extract of *Centaurea ainetensis *at 1, 3 and 10% for different time points up to 48 hr**. 1% of the extract didn't cause any considerable cell death up to 12 h where the toxicity reached only 10%, however, it caused more than 60% cell death when incubated for 24 and 48 h. At 3%, the extract was not cytotoxic up to 6 h, but caused between 15 to 20% cell death in cells treated for 8 and 12 h; and more than 80% cell death when cells were treated with plant extract for longer periods of time. At 10%, the extract was not cytotoxic up to 6 h.

The method of isolation and purification of Salograviolide A is detailed in Reference 34. Briefly, air-dried plant material soaked in methanol was filtered and the filtrate fractionated into different fractions which were bioassayed for their anti-inflammatory activities as shown in the Results Section below. Only one of the fractions was capable of mimicking the effects observed with the plant extract and was able to reverse the levels of inflammatory markers tested. This biologically active fraction was subjected to further fractionation (Figure 1A) and the resulting subfractions also bioassayed for their anti-inflammatory activities as was done above. The only subfraction that retained the biological activity was purified to give rise to the pure bioactive compound which was identified as the guaianolide, Salograviolide A (SA) (Figure 1B).

#### 2.2.2 Cell Culture

Murine intestinal epithelial cell type Mode-K cells were maintained in Dulbecco's Modified Eagle's Medium DMEM containing 1 g/l glucose and 10 mM sodium pyruvate supplemented with 10% Fetal Bovine Serum FBS, 1% non-essential amino acids and 0.5% penicillin-streptomycin. At 70-80% confluency, cells were detached by trypsinization and replated for maintenance or were used for further experiments.

#### 2.2.3 Trypan Blue Exclusion Assays

Mode-K cells were treated with different concentrations of the extract or SA for different time points. At the time of harvesting, cells were washed with phosphate buffered saline (PBS containing 137 mM NaCl, 10 mM phosphate, 2.7 mM KCl, pH 7.4) and then trypsinized and added to the supernatants. Cell suspension (50 μl) was added to 50 μl of trypan blue dye, cells were counted as either trypan positive cells (cells that were able to uptake the dye indicating dead cells) or trypan negative cells (cells that excluded the dye indicating living cells), and the percentage of dead cells was calculated.

#### 2.2.4 Western Blotting Assays

Cells were washed twice with PBS and scraped in 2× electrophoresis sample buffer (SB containing 0.25 M Tris-HCl (pH 6.8), 4% w/v SDS, 20% w/w glycerol, 0.1% bromophenol blue and protease inhibitor cocktail (40 μl/ml). Samples were then collected in microfuge tubes, boiled for 5 min, centrifuged and the supernatant representing total soluble protein extract collected and stored at -80°.

Total protein extracts were run on a 12% SDS-polyacrylamide gel and the gels were transferred to PVDF membranes overnight at 4°C. Following transfer, membranes were washed once with TPBS wash buffer (PBS containing 0.1% Tween 20) and then blocked in 5% non-fat dry milk for 2 h at room temperature. Primary antibodies were then added to the membranes and incubated for 2 h at room temperature. Unbound antibodies were washed three times with TPBS. Horse-raddish peroxidase-conjugated anti-rabbit IgG were added at 1:5000 dilution for 1 h at room temperature. Membranes were washed and incubated with Luminol reagents and directly exposed to autoradiography.

#### 2.2.5 Extraction of Nuclear Proteins

Cells were harvested and collected by centrifugation at 200 g for 10 min and washed once with PBS. Cells were lysed by rapid freezing in ethanol/dry ice and thawed by resuspension in a hypotonic ice-cold buffer containing 10 mM KCl, 1.5 mM MgCl_2_, 1 mM dithiothreitol (DTT), and 10 mM HEPES. The nuclei were centrifuged at 1250 g for 10 min at 4°C and the nuclear pellets were gently extracted in a hypertonic solution containing 0.4 mM NaCl, 1.5 mM MgCl_2_, 0.2 mM EDTA, 1 mM DTT, 0.5 mM PMSF, 20 mM HEPES and 25% glycerol, for 30 min at 4°C, and then centrifuged for 20 min at 20,000 g to collect the nuclear proteins in the supernatant. The supernatant was diluted with 30 μl of buffer containing 50 mM KCl, 20% (v/v) glycerol, 0.2 mM EDTA, 1 mM DTT, 0.5 mM PMSF and 20 mM HEPES, and stored at -80°C. Protein concentrations were determined using the Bio-Rad assay.

#### 2.2.6 Electrophoretic Mobility Shift Assay (EMSA)

NF-κB consensus oligonucleotide was end-labeled with γ-^32^P ATP using T4 polynucleotide kinase. The hybridization reaction was performed using 10-20 μg of nuclear extract, 1 μg of poly(dIdC), 1 μg of poly(dN6) as a non-specific competitor, and 10 μg of bovine serum albumin in 20 mM HEPES, 50 mM KCl, 1 mM EDTA and 5 mM DTT. The reaction was diluted with water to a v:v ratio of 1:20 of the labeled probe. The mixture was incubated for 30 min, and then stopped by adding 6 μl of 15% Ficoll solution containing the indicator dyes bromophenol blue and xylene cyanol. The reaction mixture (20 μl of each sample) was subjected to electrophoresis on a 5% non-denaturating polyacrylamide gel. The gel was transferred to Whatman filter paper, dried at 80°C under vacuum for 2 h and processed for autoradiography at -80°C overnight. The specific NF-κB band was determined by competition experiments using a mutant oligonucleotide that has lost its ability to bind to the transcription factor. Subunit specificity was determined using specific antibodies to the NF-κB components (anti Rel-A and p50) in the incubation step, which results in a supershift of the specific band due to the bound antibody [36].

#### 2.2.7 In Vivo Studies

An established rat model of ulcerative colitis induced by rectal injection of iodoacetoamide was used for *in vivo *studies to test the effects of SA in reversing and/or preventing induced inflammation [37]. In this model, the peak inflammation is reached at 24 h after iodoacetamide treatment. The *in vivo *studies were pre-approved by the "The Animal Care Program and the Institutional Animal Care and Use Committee" at the American University of Beirut (approval number 0707057).

Male rats (150-200 g) were randomly selected for treatment and control groups. Each experimental group included a group of 4-5 rats. Intestinal inflammation was induced by rectally injecting 0.1 ml of 6% iodoacetamide. SA was injected intra-peritoneal at 10 mg/kg body weight 6 h before iodoacetoamide treatment. Control rats either received SA alone as per the treated rats or ethanol (SA solvent) without any SA and/or iodoacetoamide vehicle (1% methylcellulose); while iodoacetoamide-treated rats received 10 mg/kg (*i.p. *injection) of SA twice; the first given at 6 h prior to iodoacetamide treatment and the second at the time of treatment. At the specific times scheduled rats were anesthetized with intraperitoneal pentobarbital (50 mg/kg) and intestinal tissues removed. Tissues were removed for analysis at 6, 24 and 48 h post-iodoacetoamide treatment and were examined for the degree of ulcer and for measurement of the IL-1 levels (using ELISA). An inflammation score between 0 and 3 was used to grossly evaluate the inflammation level, with 0 indicating the absence of inflammation (normal looking mucosa); 1 indicating the presence of mild inflammation (slight redness) and 3 indicating severe inflammation (includes ulceration). The *in vivo *studies were repeated three times (n = 3).

#### 2.2.8 Statistical Analysis

Data is expressed as mean ± S.D. The effectiveness of plant treatments was analyzed by one-way analysis of variance (ANOVA). Statistical probability of *P *< 0.05 was considered significant.

## 3. Results

### 3.1. Studies using Plant Extract of Centaurea ainetensis

#### 3.1.1 Cytotoxic effects of the plant extract on Mode-K cells

*The plant extract was tested for its *cytotoxicity on Mode-K cells. Trypan blue exclusion assays were performed on cells treated with different concentrations of the plant extract (1, 3 and 10%) at different time periods up to 48 h. At 10%, the plant extract was cytotoxic at 8, 12, 24 and 48 h. At 3%, the extract was cytotoxic at 12, 24 and 48 h while at 1% the extract didn't cause any considerable cell death up to 12 h. All subsequent experiments were performed using the extract at 1% for 8 and 12 h which represent the lowest concentration causing the minimal cytotoxic effects (Figure 1).

#### 3.1.2 Effect of the extract of Centaurea ainetensis on COX-2 protein levels and NF-kB activation

**Figure 2:**
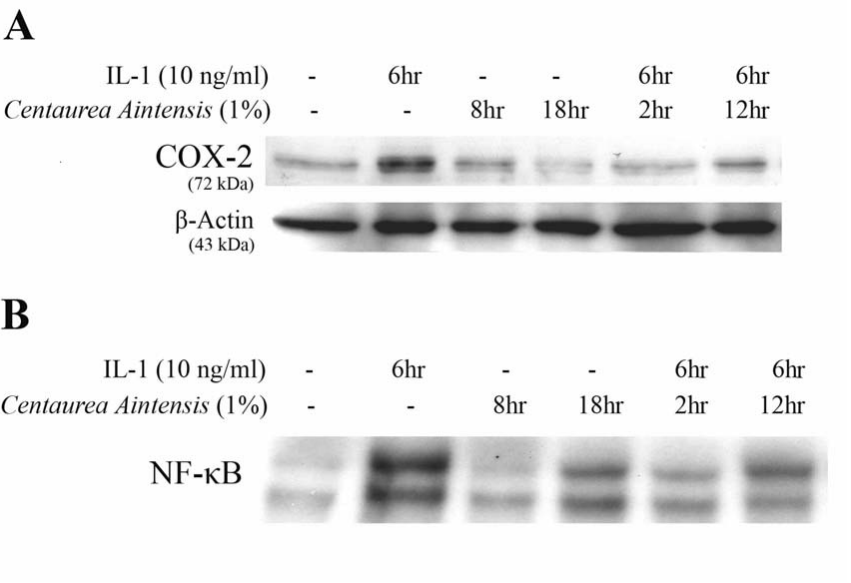
**(A) Western Blotting Analysis showing the effect of the 1% plant extract of *Centaurea ainetensis *on COX-2 protein expression in Mode-K cells**. Treatment with IL-1 for 6 h increased COX-2 protein levels (lane 2). This IL-1-induced increase was significantly inhibited by plant extract pretreatment for 2 and 12 h (lanes 5 & 6). No effect of the plant extract alone was observed (lanes 3 & 4). β-Actin was used to ensure equal protein loading. (B) Electrophoretic Mobility Shift Assay (EMSA) showing the effect of the 1% extract of *Centaurea ainetensis *on NF-κB activation in Mode-K cells in the presence and absence of IL-1 for 6 h. NF-κB was significantly activated by IL-1 treatment for 6 h (lane 2). This IL-1-induced activation was abrogated by plant extract pretreatment for 2 and 12 h (lanes 5 & 6). *Centaurea ainetensis *alone had no effect on NF-κB activation (lanes 3 & 4).

Treatment of Mode-K cells with IL-1 (10 ng/ml) caused a peak increase in COX-2 protein levels at 6 h of treatment. This increase was inhibited by pretreating cells for 2 and 12 h with the 1% plant extract. The extract alone had no effect on COX-2 protein expression as compared to the control basal levels (Figure 2A).

We have previously shown that IL-1 causes a concentration-dependent activation and translocation of NF-κB in IECs with a peak increase at 6 h, and with the predominant subunit activated being p65. To establish whether the extract has any effect on the activation and translocation of NF-κB transcription factor, electrophoretic mobility shift assay (EMSA) was performed on Mode-K cells treated with 1% of the extract, at 8 and 18 h, in the presence and absence of IL-1 (10 ng/ml). When Mode-K cells were treated with IL-1 for 6 h, a significant activation of NF-κB was detected which was abrogated upon pretreating cells for 2 and 12 h with 1% of the extract. No significant effect of the extract when used alone was observed (Figure 2B).

These results show that pretreatment of Mode-K cells with 1% of the plant extract for 2 h caused significant inhibition obtained on COX-2 protein levels as well as on NF-κB activation levels. Accordingly, further bio-guided fractionation of the extract was performed where a pure molecule, SA, was obtained and tested for its anti-inflammatory effects.

### 3.2 Studies using Salograviolide A (SA)

#### 3.2.1 Purification of SA

**Figure 3:**
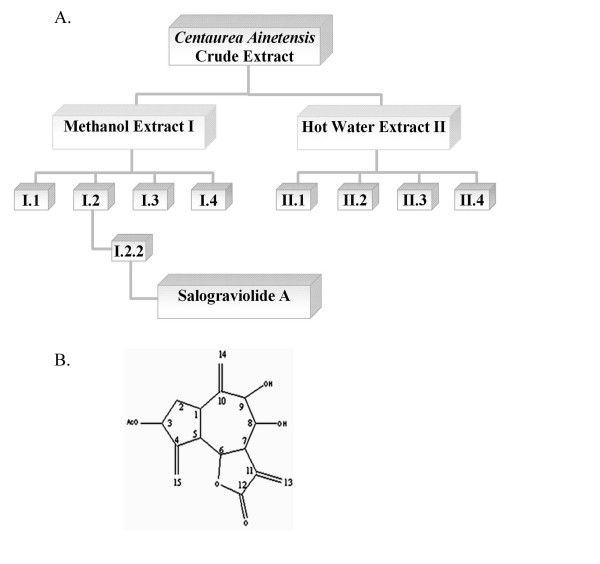
**(A) Modality used for bioguided chemical purification of Salograviolide A from *Centaurea ainetensis***. (B) Chemical structure of Salograviolide A.

The plant extract was fractionated into different fractions and each was bioassayed for its effects on IL-1-induced COX-2 and NF-κB translocation as described in the Results Section 3.1 above. Only one of the fractions was capable of mimicking the effects observed with the plant extract and was able to reverse the levels of inflammatory markers tested. This fraction was subjected to further fractionation (Figure 3A) and the resulting subfractions were also bioassayed for their anti-inflammatory activities. The only subfraction that retained the biological activity was purified to give rise to the pure bioactive compound which was identified as the guaianolide, Salograviolide A (SA) (Figure 3B).

#### 3.2.1 Cytotoxic Effects of Salograviolide A on Mode-K cells

**Figure 4:**
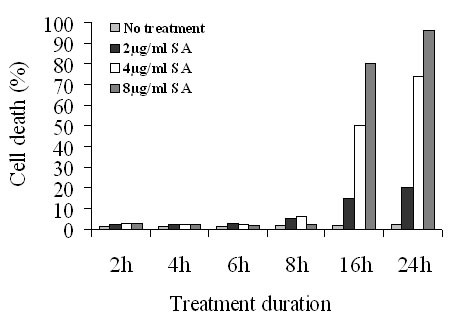
**Trypan blue exclusion assay on Mode-K cells treated with different concentrations of SA for different time periods**. SA at a concentration of 2 μg/ml only caused around 20% cell death when used at 24 h. SA at a concentration of 4 μg/ml was not cytotoxic when cells were incubated with SA for up to 8 h, but caused more than 50% cell toxicity at 16 and 24 h. At a concentration of 8 μg/ml, SA also didn't cause any significant cell death up to 8 h but caused high toxicity when cell were treated for 16 or 24 h.

In order to assess the cytotoxicity of SA on Mode-K cells, Trypan blue exclusion assays were performed on cells treated with different concentrations of SA (2, 4 and 8 μg/ml) at different time periods up to 24 h. At low concentrations, SA didn't cause any significant cell death at all time periods tested. However when used at high concentrations (4 and 8 μg/ml), it caused significant cell death (above 50%) at 12 and 24 h. SA at a concentration of 2, 4 and 8 μg/ml were used for further experiments for treatment periods not exceeding 8 h (Figure 4).

#### 3.3.2 Effect of SA on COX-2 protein expression in Mode-K cells

**Figure 5:**
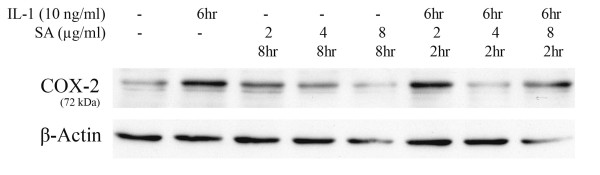
**Effect of SA on COX-2 protein expression in Mode-K cells**. Cells were pretreated with SA for 2 h prior to IL-1 6 h treatment. Total inhibition of COX-2 protein expression was observed with 4 μg/ml of SA (lane 7) as compared to cells stimulated with IL-1 alone for 6 h (lane 2). In the absence of IL-1, SA had no effect on COX-2 levels (lanes 3, 4 and 5). β-Actin was used to ensure equal protein loading.

To study the effect of SA on IL-1-induced COX-2 levels, western blotting assays were performed on total protein extracts from Mode-K cells treated with 2, 4 and 8 μg/ml of SA in the absence or presence of 10 ng/ml of IL-1 for 8 h. Treating cells with 4 μg/ml of SA for 2 h and subsequent stimulation with IL-1 for 6 h caused a significant decrease in IL-1-induced COX-2 protein levels. SA alone didn't have any effect on COX-2 protein expression as compared to the control basal levels (Figure 5). Based on these results, all further experiments were performed on cells treated with 4 μg/ml of SA for 8 h.

#### 3.3.3 Effect of SA on NF-κB activation

**Figure 6:**
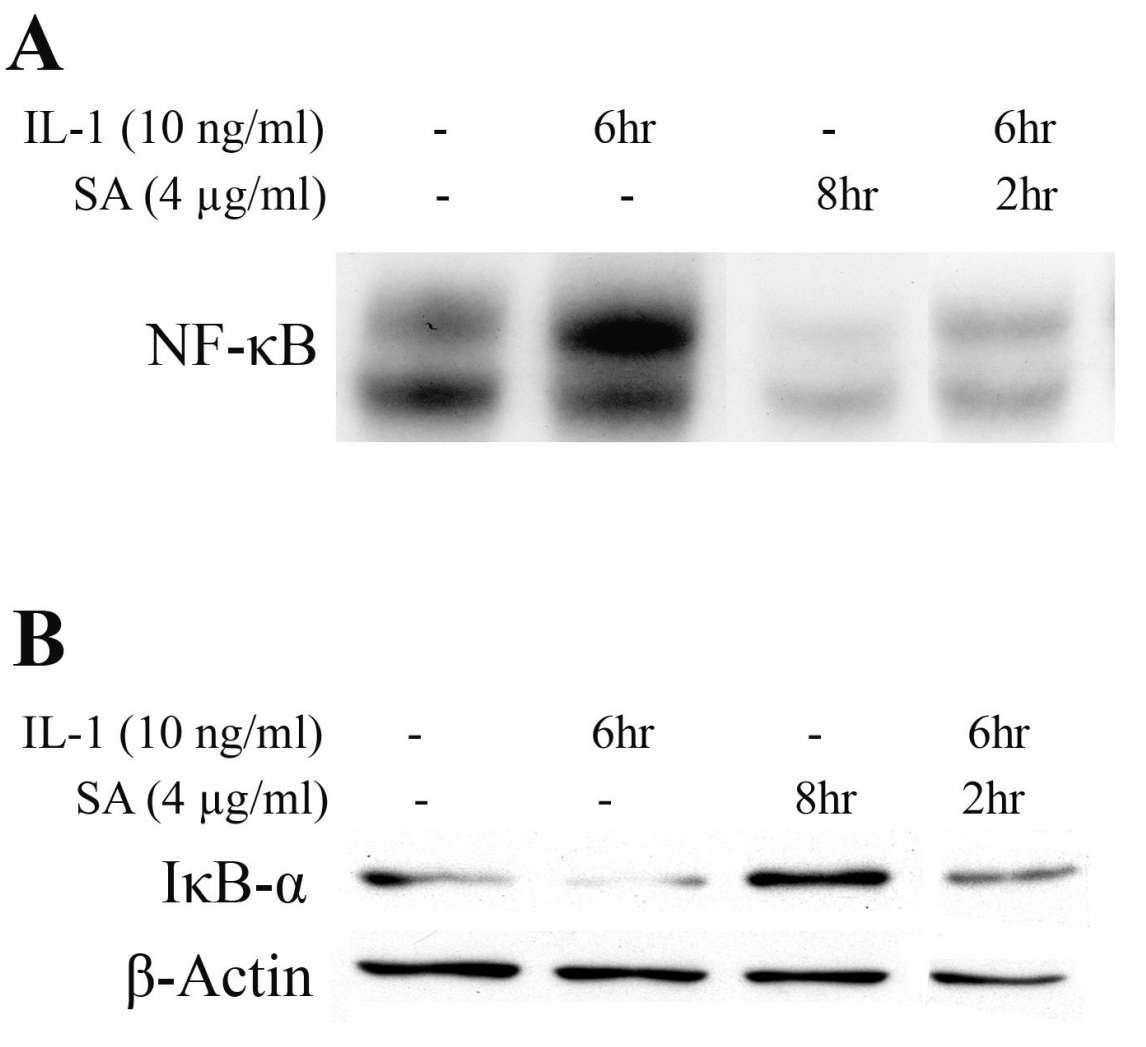
**(A) Electrophoretic Mobility Shift Assay showing the effect of SA on NF-κB activation in the presence and absence of IL-1 in Mode-K cells**. IL-1 treatment for 6 h caused activation of NF-κB (lane 2). This IL-1-induced activation was significantly inhibited by pretreating Mode-K cells with 4 μg/ml SA for 2 h (lane 4). (B) Western blotting assay showing the effect of SA on IκB-α protein expression in the presence and absence of IL-1 in Mode-K cells. IL-1 treatment for 6 h caused degradation of IκB-α protein resulting in decreased protein levels (lane 2). This IL-1-induced decrease was blocked by SA pretreament for 2 h (lane 4). No effect of SA on IκB-α protein levels was observed (lane 3). β-Actin was used to ensure equal protein loading.

To establish whether SA caused any effect on the activation and translocation of NF-κB transcription factor, electrophoretic mobility shift assay (EMSA) was performed on nuclear extracts of Mode-K cells treated with 4 μg/ml of SA in the presence and absence of IL-1 (10 ng/ml). IL-1 treatment for 6 h caused significant activation of NF-κB transcription factor. This activation was significantly inhibited by 4 μg/ml of SA pretreatment for 2 h. SA alone didn't show any effect on NF-κB levels (Figure 6A).

In an attempt to find the part of the NF-κB activation cascade that is influenced by SA, the effect of SA on IκB protein levels was assessed. The IL-1 induced degradation of IκB-α was found to be inhibited by SA pretreatment for 2 h at a concentration of 4 μg/ml (Figure 6B).

#### 3.3.4 Effect of SA on colonic inflammation in a rat model of IBD

SA treatment did not cause any inflammation or change in inflammatory cytokines similar to what is usually observed in control untreated, or vehicle-treated rats. Rats treated twice with SA prior to iodoacetamide showed significantly lower inflammation scores than rats treated with iodoacetamide alone at 6 and 24 h post-treatment (Table 1).

**Table 1:** Effect of SA on reversing inflammation in the in-vivo IBD model

**Treatment**	**Tissues Removed at 6 h After Treatment^a^**	**Tissues Removed at 24 h After Treatment^a^**
**Control (no treatment)**	0	0

**Treatment with SA (12 mg/kg, i.p. injection)**	0	0

**Rats treated with ethanol**	0	0

**Rats treated with methyl cellulose**	0	0

**Rats treated with iodoacetoamide (representing inflamed tissue)**	2	3^b^

**Rats treated twice with SA prior to iodoacetoamide**	1.0	1.0^c^

^a ^Inflammation score

^b ^P < 0.005, ^b ^represents the comparison of iodoacetoamide treatment on inflammation score as compared to untreated control. Data are means of 3 separate experiments.

^c ^P < 0.005, ^c ^represents the comparison of SA pretreatment on iodoacetoamide treated rats as compared to iodoacetoamide treated rats. Data are means of 3 separate experiments.

Levels of IL-1 in colonic tissue were shown to be significantly lower in rats treated with SA prior to iodoacetamide treatment (Table 2). These results suggest that SA may act as preventive means to reduce inflammation in an *in vivo *model.

**Table 2:** Effect of SA on IL-1 Levels in Intestinal Tissue in an in-vivo

**Treatment**	**Levels of IL-1 (ng/ml) in Colonic Tissues Removed 6 h After Treatment**	**Levels of IL-1 (ng/ml) in Colonic Tissues Removed 24 h After Treatment**
**Rats treated with SA alone** **(control, n = 3)**	8.9 ± 1.9	12.1 ± 0.3

**Rats treated with iodoacetoamide (representing inflamed tissue, n = 3)**	27.3^a^± 3.0	18.6 ± 0.6

**Rats treated with SA prior to iodoacetoamide treatment** **(represents recovered tissue, n = 3)**	16.6^b^± 0.2	11.4 ± 1.4

^a ^P < 0.005, ^a ^P value represents the comparison of IL-1 levels in iodoacetoamide treated tissue as compared to untreated control. Data are means ± SE of 3 separate experiments.

^b ^P < 0.05, ^b ^P value represents the comparison of SA pretreatment on IL-1 levels in iodoacetoamide treated rats as compared to iodoacetoamide treated rats. Data are means ± SE of 3 separate experiments.

## 4. Discussion

In the present study the effects of the *Centaurea ainetensis *extract and SA on COX-2 expression and NF-κB translocation in an intestinal epithelial cell model (Mode-K cells) of inflammation and SA's ability to reverse and/or prevent inflammation in a rat model of IBD were studied. COX-2, a 72 kDa protein, which is not usually present in IECs under normal conditions, is significantly induced by IL-1 treatment [38] with peak expression reached 6 h post treatment [39]. SA caused significant inhibition on IL-1-induced COX-2 protein levels as well as on NF-κB activation levels. Under basal conditions, NF-κB, a transcription factor, is maintained in an inactive form by IκB inhibitory proteins. Many inflammatory stimuli lead to the proteolytic degradation of IκB proteins [40] thus freeing NF- κB to translocate to the nucleus. The effect caused by SA on NF-κB was found to be at least partially due to inhibition of IL-1 induced degradation of IκB-α. In addition to the SA anti-inflammatory effects observed in the *in vitro *studies, SA was found to significantly reduce inflammation in an *in vivo *model of IBD.

Many of the anti-inflammatory activities of some medicinal plants have been attributed to their contents of sesquiterpene lactones (SLs). The isolated active compound from *Centaurea ainetensis *Salograviolide A was identified as a guaianolide belonging to the sesquiterpene lactone family. The mechanism of action of SLs against inflammation has been extensively investigated and, based on these studies as well as the efficacy of SLs in modulating inflammation in response to a variety of stimuli [20,41-45], there are strong indications that all SLs act through a common step beyond the point of integration of different signals. This general mechanism of action can be seen as a dual mechanism that eventually leads to the same endpoint: a decrease in NF-κB DNA binding activity. Previous reports have suggested that the main mode of action of SLs is through inhibiting the induced degradation of IκB proteins α and β [43,46] by possibly inhibiting IκB Kinase (IKK), a kinase that phosphorylates and tags IκB proteins for destruction [47]. However, other studies have shown that a direct interaction between SLs and p65 (Rel A) in NF-κB resulting in a direct inhibition of DNA binding is the main mechanism of action of SLs [44,48]. Our results confirm this dual mechanism of action: we have shown that SA inhibited IL-1-induced IκB-α degradation leading to a decreased NF-κB activity. SA also caused a decrease in NF-κB activity when cells were treated after IL-1 treatment; that is after the induced degradation of IκB proteins, suggesting that SA may be acting directly on the translocated NF-κB preventing it from binding to DNA. In both cases, given the importance of NF-κB in promoting the expression of numerous pro-inflammatory genes including those encoding the enzymes COX-2 and iNOS [49], causing decreases in their products PGE_2 _[15] and NO [17], and providing a possible mechanism for the observed decrease caused by SA on IL-1-induced COX-2 protein expression.

The biological activity of SA could be due to the presence of α,β-unsaturated carbonyl structures mainly the α-methylene-γ-lactone. These structures have the ability to react with nucleophiles by a Michael-type addition [50] which consequently can react irreversibly with sulfhydryl groups in the cell including those found on cysteine [27]. Specifically, in NF-κB/p65, cysteine 38 that participates in DNA binding by forming a hydrogen bond with the sugar/phosphate backbone of the κB-DNA could be the site of alkylation by SA and consequent direct inhibition of NF-κB DNA binding [48,51]. Moreover, the activation loop of the catalytic site of IκB Kinase (IKK) contains a critical cysteine, cysteine-179 [52,53], that can react with Michael donors explaining how SA can cause inhibition of IκB protein degradation [48]. The number of α,β-unsaturated carbonyl structures capable of undergoing a Michael addition, has been recognized as a major factor in determining the potency of SLs [51]. Despite having only one alkylating center, SA has a very high potency when compared to the ability of other bifunctional SLs in inhibiting the transcription factor NF-κB. The lower inhibitory concentration of SA may be due to the presence of a hydroxyl group capable of forming a hydrogen bond near the alkylating structure and consequently stabilize the covalent binding. However, SA has another hydroxyl group and, quantitative structure-activity relationship studies specific to guaianolides correlate an increasing number of hydroxyl groups with a decrease in NF-κB inhibition activity [54], an effect which could be offset by enhancing the rate of cysteine addition in the presence of an O-acyl group.

## 5. Conclusion

This is the first report of the anti-inflammatory activity of Salograviolide A isolated from *Centaurea ainetensis *in both *in vitro *and *in vivo *models of inflammation. In addition to disclosing the mechanism of action of this SL *in vitro*, our study highlights once again the important role played by NF-κB in intestinal inflammation. Optimizing the number of hydroxyl group in this molecule might make SA a valuable molecule for the development of natural therapies for inflammatory diseases through enhancing its potency and at the same time reducing the risk of unwanted side effects.

## 6. List of Abbreviations

APS: Ammonium Persulfate; BSA: Bovine Serum Albumin; COX-2: Cyclooxygenase-2; DMEM: Dulbecco's Modified Eagle's Medium; EMSA: Electrophoretic Mobility Shift Assay; EDTA: Ethylenediamine Tetraacetic Acid; FBS: Fetal Bovine Serum: HRP: Horse Radish Peroxidase; IBD: Inflammatory Bowel Disease; IKK: Inhibiting IκB Kinase; IκB: Inhibitory Subunit; IL-1: Interleukin; IEC: Intestinal Epithelial Cell; LOX: 5-Lipoxygenase; NO: Nitric Oxide; NF-κB: Nuclear Factor-κB; PVDF: Polyvinylidene Difluoride; PGE_2_: Prostaglandin E_2_; SA: Salograviolide A; SLs: Sesquiterpene lactones; SDS: Sodium Dodecyl Sulfate; TEMED: N,N,N',N',Tetramethylethylenediamine; TNF-α: Tumor Necrosis factor-Alpha.

## 7. Competing interests

The authors declare that they have no competing interests.

## 8. Authors' Contributions

JAS and RAA made substantial contributions to data acquisition, NAS, RST and FRH made substantial contributions to analysis and interpretation to conception and design, RS, JAS and RAA made contributions in data analysis and drafting the manuscript and revising it critically for important intellectual content; and FRH have given final approval of the version to be published. All authors read and approved the final manuscript

## Pre-publication history

The pre-publication history for this paper can be accessed here:


